# A Memorable Moment During Laparoscopic Cholecystectomy: A Case Report

**DOI:** 10.7759/cureus.4520

**Published:** 2019-04-22

**Authors:** Mohamed Ahmed, Rasha Saeed, Ahmed Mahmoud, Talat A Attar, Amarseen Mikael

**Affiliations:** 1 Surgery, Riverside Community Hospital, Riverside, USA; 2 Surgery, Arrowhead Regional Medical Center, Fontana, USA

**Keywords:** ercp, biliary stent, acute cholecystitis, cholengitis

## Abstract

Common bile duct (CBD) stones are encountered in 14%-15% of patients with symptomatic gall stone disease. Endoscopic retrograde cholangiopancreatography (ERCP) is a primary modality for the management of pancreaticobiliary disorders. We present a case of unintentional stent placement into the gall bladder discovered during surgery.

## Introduction

Common bile duct (CBD) calculi are found in 15% of patients with chronic cholecystitis and 14.2% with acute inflammation of the gallbladder [1]. Early endoscopic retrograde cholangiopancreatography (ERCP) is associated with lower in-hospital and 30-day mortality in patients with cholangitis [2]. The incidence rate of the procedure complications is 7% [3]. It includes infection, pancreatitis, hemorrhage, and perforation can occur even in expert hands [4]. In our case, unintentional stent cannulation of the cystic duct and gall bladder led to a memorable moment during laparoscopic cholecystectomy.

## Case presentation

**Figure 1:**
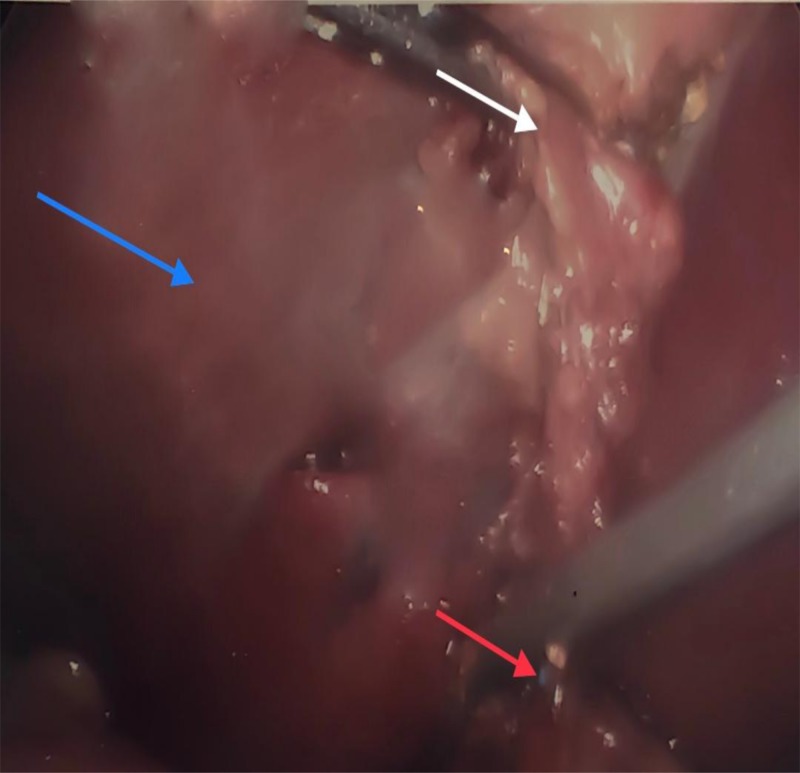
The misplaced stent into the cystic duct became evident as the duct was incised to retrieve presumed stones and that was a memorable moment as a common bile duct injury was the concern (stent is usually introduced into the common bile duct and not the cystic duct). Red arrow pointing to a blue stent initially thought to be in the common bile duct and was later found to have been misplaced into the cystic duct; blue arrow pointing to the liver; white arrow pointing to the gall bladder

A 40-one-year-old morbidly obese (body mass index 57.5 kilograms/square meter) female patient presented to our emergency room with worsening epigastric abdominal pain and fever of eight days duration. The liver function test was mildly elevated. MRCP revealed the dilation of the common bile duct with an abrupt truncation of the distal common bile duct consistent with choledocholithiasis. ERCP with the extraction of multiple stones and stent placement was performed. During laparoscopic cholecystectomy, hard areas were felt in the cystic duct and were presumed to be stones. The stent came into view when a small incision was made in the cystic duct to retrieve the presumed stones and a common bile duct (CBD) injury was suspected (Figure 1).

**Figure 2:**
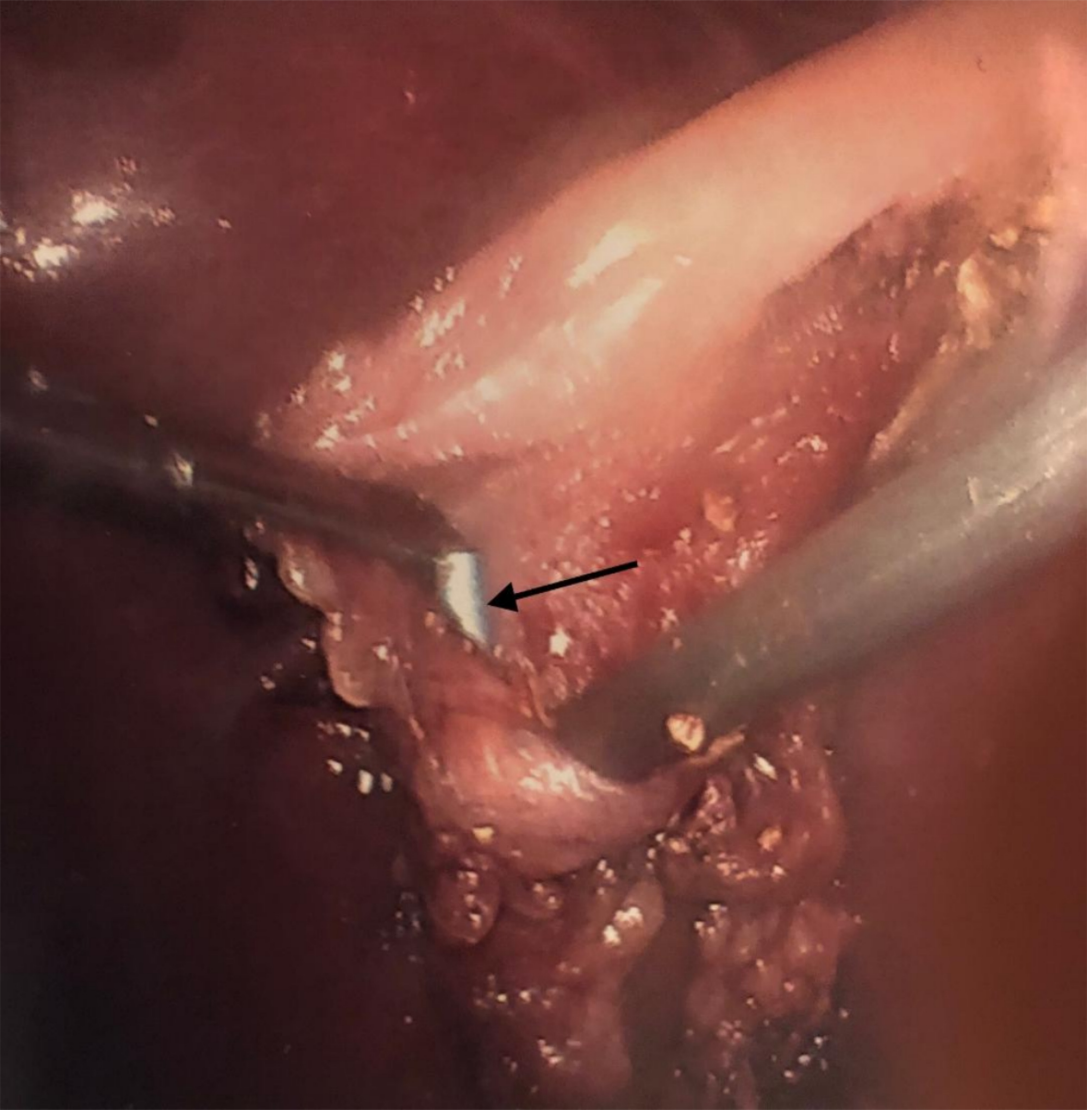
Gall bladder opened showing where the stent terminated White arrow pointing toward a blue stent

Dissection of the gall bladder off the liver bed was not fruitful in defining anatomy with absolute certainty. The gall bladder was opened to visualize the cystic duct internal opening. The stent appeared to terminate in the gall bladder and an intraoperative cholangiogram was felt not to be needed at this point (Figure 2).

The stent was retrieved and cholecystectomy completed.

## Discussion

In September 1985, Erich Muhe performed the first laparoscopic cholecystectomy [5]. The surgical community was skeptical of his new operation. Since then, it had become the gold standard treatment for gallstone disease [6]. The incidence rate of common bile duct injury 0.5% [7]. The management of patients following major bile duct injury is a surgical challenge requiring the skills of an experienced hepatobiliary surgeon at tertiary referral center [8]. Laparoscopic techniques play a role in the management of CBD injuries at the initial operation to confirm and possibly repair the suspected injury. A small defect bile leak can be managed with laparoscopic repair, drainage of the area, and ERCP [9]. A severe CBD injury requires bilioenteric anastomosis in the form of hepaticojejunostomy or hepaticoduodenostomy, which had shown good long-term results [10]. Patients presenting with a symptomatic leak after surgery, re-laparoscopy to identify the source, and drainage of intra-abdominal collection, excluding injury to surrounding structures, guide the decision between conservative management and definitive repair [11].

## Conclusions

Reviewing ERCP films prior to surgery, when available, is always helpful. The management of patients following major bile duct injury is a surgical challenge requiring the skills of an experienced hepatobiliary surgeon. In our case, a laparoscopic attempt to better identify anatomy proved to be rewarding.

## References

[1] Coelho J, Buffara M, Pozzobon C, Altenburg FL, Artigas GV (1984). Incidence of common bile duct stones in patients with acute and chronic cholecystitis. Surg Gynecol Obstet.

[2] Mulki R, Shah R, Qayed E (2019). Early vs late endoscopic retrograde cholangiopancreatography in patients with acute cholangitis: a nationwide analysis. World J Gastrointest Endosc.

[3] Andriulli A, Loperfido S, Napolitano G (2007). Incidence rates of post-ERCP complications: a systematic survey of prospective studies. Am J Gastroenterol.

[4] Szary M, Al-Kawas F (2013). Complications of endoscopic retrograde cholangiopancreatography: how to avoid and manage them. Gastroenterol Hepatol.

[5] Sicklick J, Camp M, Lillemoe K (2005). Surgical management of bile duct injuries sustained during laparoscopic cholecystectomy: perioperative results in 200 patients. Ann Surg.

[6] Muhe E (1991). Laparoscopic cholecystectomy: late results [Article in German]. Langenbecks Arch Chir Suppl Kongressbd.

[7] Kohn J, Trenk A, Kuchta K (2018). Characterization of common bile duct injury after laparoscopic cholecystectomy in a high-volume hospital system. Surg Endosc.

[8] Melton G, Lillemoe K, Cameron J, Sauter PA, Coleman J, Yeo CJ (2002). Major bile duct injuries associated with laparoscopic cholecystectomy: effect of surgical repair on quality of life. Ann Surg.

[9] Barband A, Jorgensen J, Hunt J (2011). Relaparoscopy in minor bile leakage after laparoscopic cholecystectomy: an alternative approach?. Surg Laparosc Endosc Percutan Tech.

[10] Gupta V, Jayaraman S (2017). Role for laparoscopy in the management of bile duct injuries. Can J Surg.

[11] Wills V, Jorgensen J, Hunt D (2000). Role of relaparoscopy in the management of minor bile leakage after laparoscopic cholecystectomy. Br J Surg.

